# Exploration of m^6^A methylation regulators as epigenetic targets for immunotherapy in advanced sepsis

**DOI:** 10.1186/s12859-023-05379-w

**Published:** 2023-06-17

**Authors:** Weiwei Qian, Jian Zhou, Songtao Shou

**Affiliations:** 1 Tianjin Medical University, Tianjin, 300203 China; 2 Department of Emergency, Shangjin Nanfu Hospital, West China Hospital, Sichuan University, Chengdu, 610044 Sichuan China; 3 Department of Immunology, International Cancer Center, Shenzhen University Health Science Center, Shenzhen, 518060 China; 4 Department of Emergency, Tianjin Medical University General Hospital, 154 Anshan Road, Heping District, Tianjin, 300052 China

**Keywords:** Advanced sepsis, m^6^A methylation modification, Immunoregulation

## Abstract

**Background:**

This study aims to deeply explore the relationship between m^6^A methylation modification and peripheral immune cells in patients with advanced sepsis and mine potential epigenetic therapeutic targets by analyzing the differential expression patterns of m^6^A-related genes in healthy subjects and advanced sepsis patients.

**Methods:**

A single cell expression dataset of peripheral immune cells containing blood samples from 4 patients with advanced sepsis and 5 healthy subjects was obtained from the gene expression comprehensive database (GSE175453). Differential expression analysis and cluster analysis were performed on 21 m^6^A-related genes. The characteristic gene was identified based on random forest  algorithm, and the correlation between the characteristic gene METTL16 and 23 immune cells in patients with advanced sepsis was evaluated using single-sample gene set enrichment analysis.

**Results:**

IGFBP1, IGFBP2, IGF2BP1, and WTAP were highly expressed in patients with advanced sepsis and m^6^A cluster B. IGFBP1, IGFBP2, and IGF2BP1 were positively correlated with Th17 helper T cells. The characteristic gene METTL16 exhibited a significant positive correlation with the proportion of various immune cells.

**Conclusion:**

IGFBP1, IGFBP2, IGF2BP1, WTAP, and METTL16 may accelerate the development of advanced sepsis by regulating m^6^A methylation modification and promoting immune cell infiltration. The discovery of these characteristic genes related to advanced sepsis provides potential therapeutic targets for the diagnosis and treatment of sepsis.

**Supplementary Information:**

The online version contains supplementary material available at 10.1186/s12859-023-05379-w.

## Background

Sepsis is a life-threatening syndrome caused by a deregulated host response to bacterial, fungal, or viral infection, involving physiological, pathological, and biochemical abnormalities [1, 2]. It is generally believed that the symptoms of early sepsis are mild, manifested as non-specific symptoms such as fever, headache, and fatigue. However, physical examination may reveal inflammatory reactions and symptoms of redness and swelling at the site of local infection. Over time, the condition may further worsen, with severe symptoms such as high fever, chills, shortness of breath, and increased heart rate. Advanced sepsis typically manifests as multiple organ dysfunction syndrome (MODS), which involves multiple organ dysfunction and severe symptoms such as renal insufficiency, pneumonia, myocardial injury, and bleeding. At this time, the condition is already very critical and requires urgent treatment [3]. In advanced sepsis, the number and function of various peripheral immune cells, including CD4 + T cells, CD8 + T cells, B cells, and natural killer cell, may be affected, resulting in the inability of the immune system to effectively eliminate pathogens [4]. Therefore, analyzing the expression of peripheral immune cells in patients with advanced sepsis can provide detailed information on the immune response to sepsis and help to gain a deeper understanding of the specific molecular mechanisms underlying the high mortality rate caused by this disease.

N6 methyladenosine (m^6^A) is the most prevalent post-transcriptional chemical modification on RNA molecules, which participates in the regulation of various biological processes including immunity, metabolism, proliferation, and apoptosis, accounting for more than 60% of all RNA epigenetics [5]. The biological function of m^6^A modification is dynamically and reversibly mediated by methyltransferases (writers), demethylases (erasers), and m^6^A recognition proteins (readers), which is involved in the occurrence and development of various diseases and is also related to the high heterogeneity in advanced sepsis [6–8]. We speculate that m^6^A modification plays an important role in immune regulation in advanced sepsis. In the present study, bioinformatics methods were used to analyze the differential expression patterns of m^6^A methylation regulators and explore their relationship with immune cell infiltration, aiming to provide a theoretical basis for the individualized risk determination and treatment target selection of advanced sepsis.

## Methods

### Source of data

The dataset GSE175453 containing the transcriptome data of peripheral blood mononuclear cells (PBMCs) in the blood samples from 4 patients with advanced sepsis (14–21 days after sepsis) and 5 healthy subjects was obtained from the Gene Expression Omnibus (GEO) [9].

### Screening of m^6^A methylation regulators

A total of 21 m^6^A-related genes were obtained from the literature to identify their different m^6^A modification modes: RNA binding motif protein 15B (RBM15B), insulin-like growth factor binding protein 3 (IGFBP3), IGFBP1, IGFBP2, insulin-like growth factor 2 mRNA binding protein 1 (IGF2BP1), zinc finger CCCH domain-containing protein 13 (ZC3H13), Casitas B-lineage lymphoma-transforming sequence-like protein 1 (CBLL1), YTH N^6^-methyladenosine RNA binding protein 1 (YTHDF1), YTHDF2, heterogeneous nuclear ribonucleoprotein C (HNRNPC), embryonic lethal-abnormal vision like protein 1 (ELAVL1), methyltransferase-like 3 (METTL3), RNA-binding motif protein X-linked (RBMX), leucine-rich pentatricopeptide repeat (PPR)-motif-containing protein (LRPPRC), METTL16, fat mass and obesity-associated gene (FTO), Wilms tumor 1-associated protein (WTAP), YTH domain containing 1 (YTHDC1), fragile X mental retardation type 1 (FMR1), RBM15, and Heterogeneous nuclear ribonucleoprotein A2B1 (HNRNPA2B1).

### Analysis of sepsis characteristic genes based on random forest (RF) algorithm

The m^6^A methylation regulatory factors differentially expressed in peripheral blood immune cells between healthy subjects and advanced sepsis patients were analyzed using the “limma” R package, with the screening criteria of P < 0.05 [10]. Pearson correlation coefficient was used to analyze the correlation of the differentially expressed m^6^A methylation regulators (r > 0.3, P < 0.05). The upport vector machine (SVM) algorithm and RF algorithm were performed using the “randomForest” R package to construct a model for predicting the occurrence of advanced sepsis [11, 12]. The prediction accuracy of the model was evaluated using the residual boxplot, the residual reverse cumulative distribution, and the receiver operating characteristic (ROC) curve.

### m^6^A clustering and immunocyte infiltration analysis

Using the “ConsensusClusterPlus” R package, the m^6^A clusters were identified by consensus clustering based on the m^6^A methylation regulatory factors [13]. Then, the abundance of 23 immune cells in advanced sepsis was evaluated using single-sample gene set enrichment analysis (ssGSEA) to further investigate the correlation [14–17].

### Statistical analysis

Statistical analysis was performed using R (version 4.1.0). Linear regression analysis and Pearson correlation coefficient (r) were used to determine the correlation between gene expression patterns. Nonparametric one-way analysis of variance (ANOVA) was used to compare the variables between different groups. The comparison between two groups was performed using the t-test. A value of P < 0.05 was indicative of statistical significance.

## Results

### Differentially expressed m^6^A methylation regulators in peripheral immune cells in advanced sepsis

**Fig. 1:**
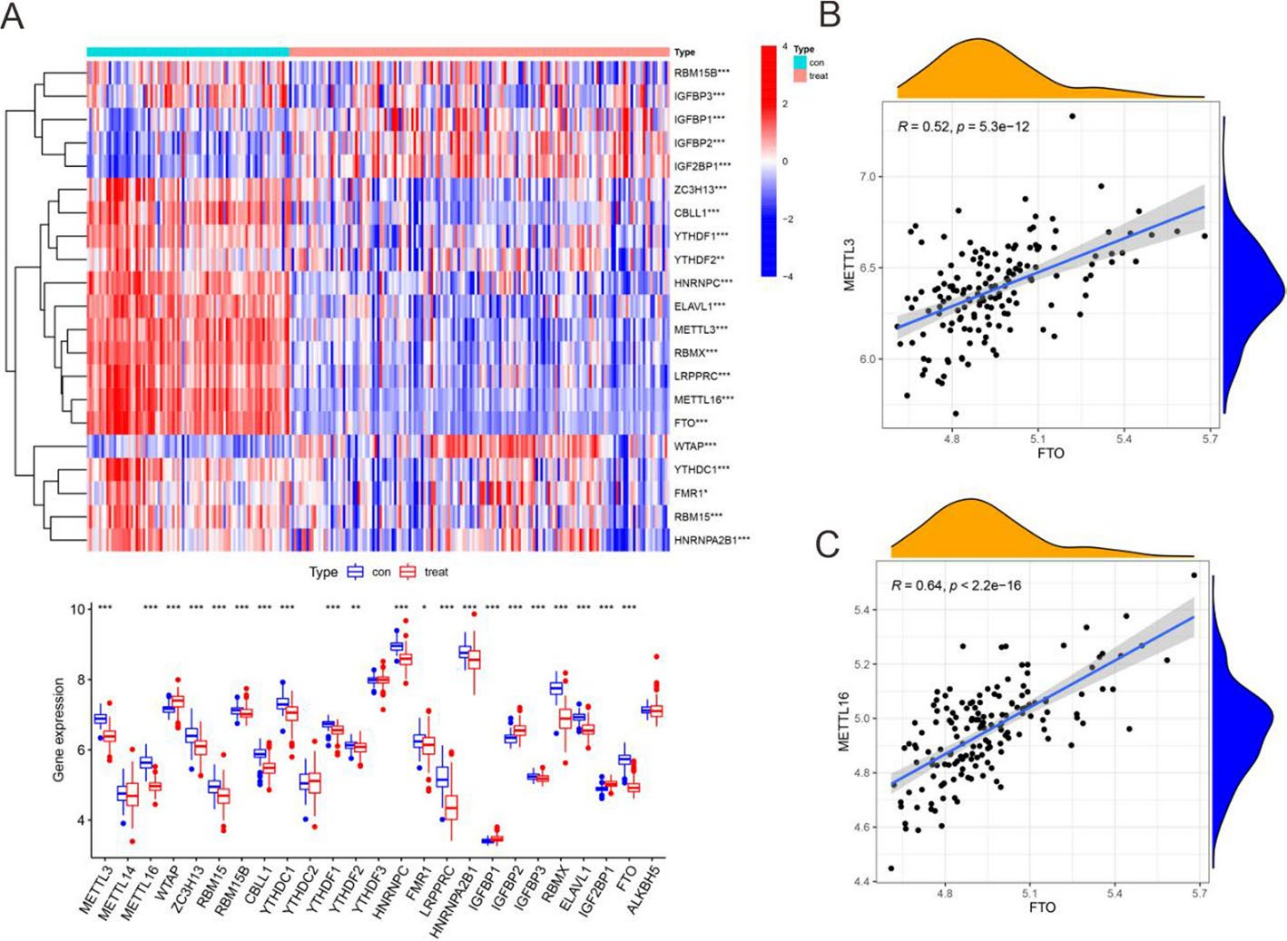
Differential expression of m^6^A methylation regulatory factors in sepsis. (**A**) Heatmap analysis of differentially expressed m^6^A methylation regulators; (**B**) Correlation analysis of gene expression between METTL3 and FTO; (**C**) Correlation analysis of gene expression between METTL16 and FTO. Con: control patients; treat: sepsis patients

There were 21 differentially expressed m^6^A methylation regulators in peripheral immune cells between healthy subjects and advanced sepsis patients in the GSE175453 dataset. As shown in Fig. 1A, IGFBP1, IGFBP2, IGF2BP1, and WTAP were highly expressed in advanced sepsis patients, and the remaining 17 regulators were poorly expressed. Further analysis showed that there was a positive correlation between the expression of METTL3 (Fig. 1B), METTL16 (Fig. 1C), and FTO. These results suggested that m^6^A methylation might play an important role in sepsis.

### Identification of two m^6^A clusters

**Fig. 2:**
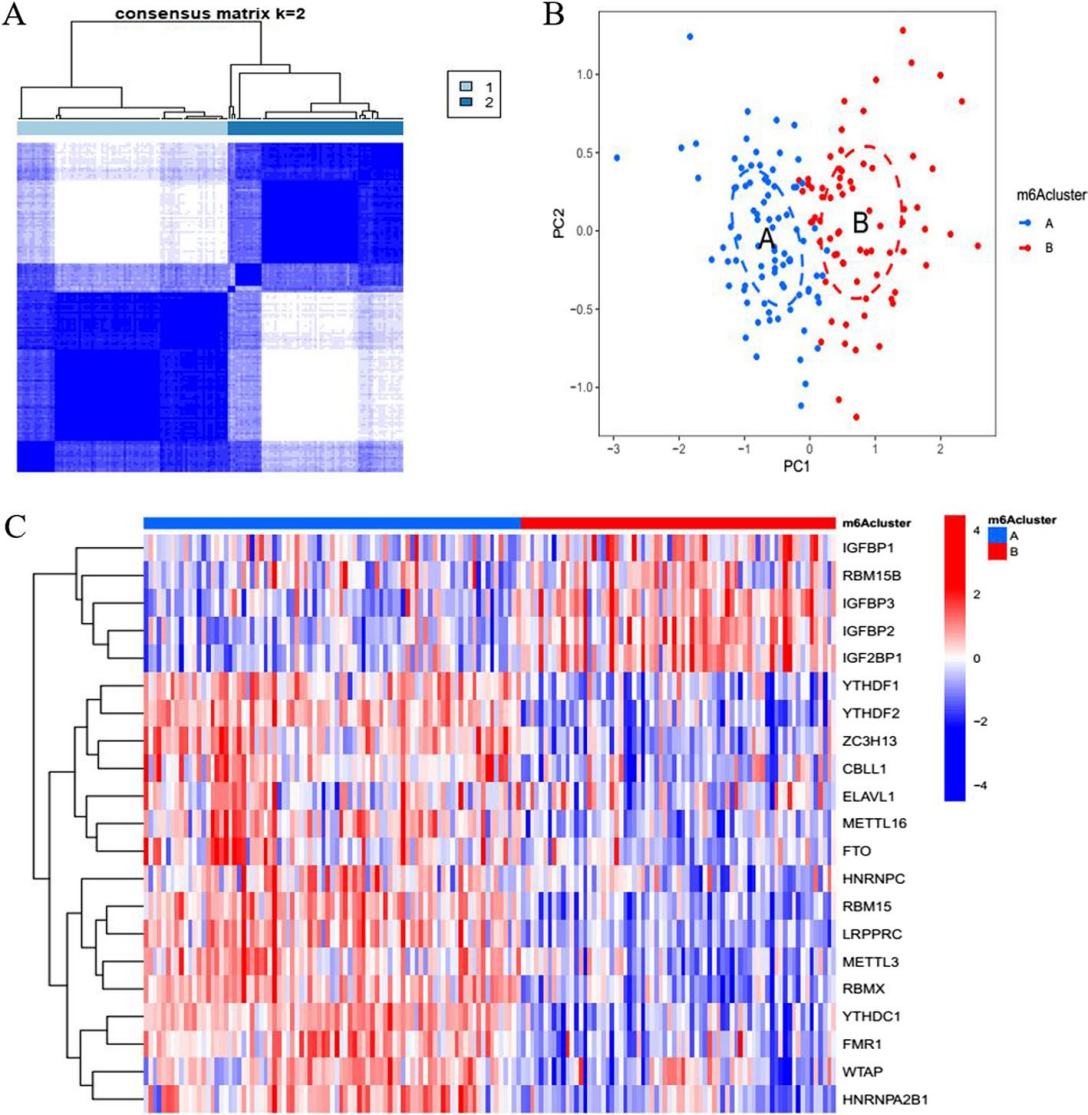
Two different m^6^A clusters. **A** Consensus clustering matrix of sepsis samples, k = 2; **B** Principal component analysis of two different m^6^A clusters; **C** Heatmap of differentially expressed genes in two m^6^A clusters

Consensus cluster analysis was performed using the “ConsensusClusterPlus” R package based on differential m^6^A methylation regulators, and two m^6^A clusters (m^6^A cluster A and m^6^A cluster B) were identified (Fig. 2A). The results of principal component analysis (PCA) showed that m^6^A RNA methylation regulators could be classified into two m^6^A clusters (Fig. 2B). Figure 2 C shows the heat map of the differentially expressed genes in two m^6^A clusters (Additional file 1: Table S1). IGFBP1, IGFBP2, and IGF2BP1 were highly expressed in cluster B (Additional file 2: Table S2).

### Construction of sepsis prediction model based on RF algorithm

**Fig. 3:**
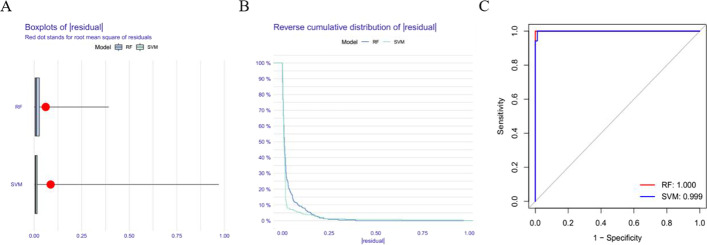
The prediction model of sepsis constructed by random forest (RF). **A** Boxplots of residual distribution; **B** Residual inverse cumulative distribution; **C** Receiver operating characteristic curve

The performance of SVM algorithm and RF algorithm was compared using residual boxplots (Fig. 3A), residual reverse cumulative distribution (Fig. 3B), and ROC curve (Fig. 3C). The results showed that there was no significant difference between the two algorithms. Compared with SVM algorithm, RF algorithm had higher accuracy, so the subsequent screening of disease characteristic genes was based on RF algorithm.

### Immunocyte infiltration analysis

**Fig. 4:**
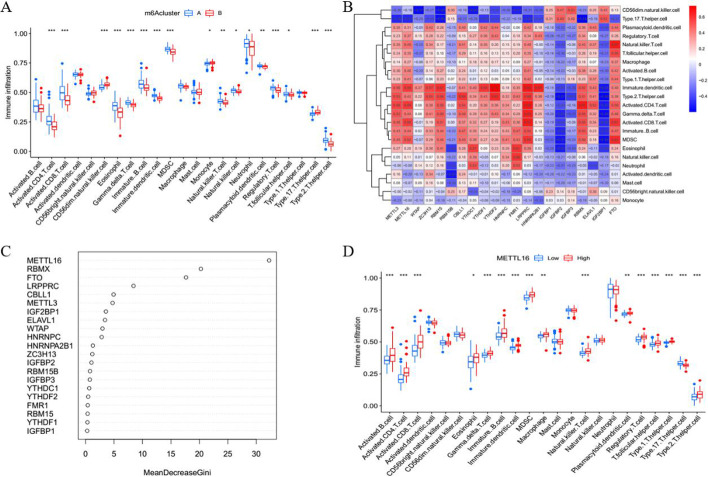
Immune cell infiltration analysis. **A** Boxplots showing the infiltration of immune cells in two m^6^A clusters; **B** Correlation heatmaps of 21 m^6^A methylation regulatory factors and immune cell infiltration by single sample gene cluster enrichment analysis (ssGSEA); **C** Importance of 21 m^6^A regulatory factors based on random forest (RF) model; **D** Positive correlation between METTL16 and immune cell infiltration

Due to the fact that a large number of immune cells are involved in sepsis, we further analyzed the difference in immune cell infiltration between the two m^6^A clusters. The activated CD4+T cells, activated CD8 + T cells, regulatory T cells, Th2 helper T cells, and Th17 helper T cells showed significant differences between the two m^6^A clusters (Fig. 4A). In addition, ssGSEA was used to determine the correlation between the expression of 21 m^6^A methylation regulatory factors and the infiltration of immune cells (Fig. 4B). Correlation heat map analysis showed that IGFBP1, IGFBP2, and IGF2BP1 had a significant positive correlation with Th17 helper T cells. Based on the RF model, the genetic importance of 21 m^6^A methylation regulators was ranked (Fig. 4C). Then we selected METTL16 gene to further observe its correlation with immune cell infiltration, and found that it was significantly positively correlated with activated CD4+T cells, activated CD8 + T cells, natural killer T cells, regulatory T cells, Th1 helper T cells, and Th2 helper T cells (Fig. 4D).

## Discussion

Advanced sepsis is a serious life-threatening infectious disease, and its pathological and physiological mechanisms are still not fully understood [18, 19]. m^6^A regulatory factors are closely related to human diseases, and abnormal m^6^A methylation modification is believed to mediate various immune responses and the pathogenesis of autoimmune related diseases [20]. According to the analysis of m^6^A-single nucleotide polymorphism (SNP) and expression quantitative trait locus (eQTL) dataset, m^6^A is involved in regulating bacterial infection. Sun et al. identified 1321 genes as the sites of m^6^A cis-eQTL [21]. These genes are highly enriched in the pathways involving platelet degranulation and staphylococcus aureus infection, which are crucial to the pathophysiological process of sepsis. In addition, multiple studies have reported the typing role of m^6^A regulatory factors in sepsis and their correlation with immune cells [8, 22]. These findings provide convincing evidence for the correlation between m^6^A modification and sepsis episodes.

In recent years, the development of single-cell RNA seq technology has provided a novel approach for sepsis research [9, 23]. Compared with traditional gene expression analysis methods, single cell RNA seq can analyze the transcriptome of a single cell, find more cell types and subtypes, and analyze heterogeneous cells more accurately. Researchers have found unique transcriptome patterns of multiple circulating immune cell subtypes, including B- and CD4+, CD8+, activated CD4+, and activated CD8+T lymphocytes, as well as NK, NKT, and plasma cell like dendritic cells in patients with advanced sepsis [9]. However, the specific molecular mechanisms leading to these changes remain unclear, and the therapeutic targets have not been determined yet.

This study focused on analyzing the relationship between m^6^A methylation modification and multiple lymphocytes in patients with advanced sepsis, and explored potential epigenetic therapeutic targets. In this study, the ssGSEA method was used to evaluate the abundance of 23 immune cells in advanced sepsis. The results showed that IGFBP1, IGFBP2, IGF2BP1, and WTAP were highly expressed in patients with advanced sepsis and m^6^A cluster B; IGFBP1, IGFBP2, and IGF2BP1 showed a significant positive correlation with Th17 helper T cells. These results all suggested that m6A methylation played a crucial role in immune regulation in advanced sepsis.

It is well-known that RF is a widely used machine-learning algorithm in bioinformatics, which has been proven to be effective in identifying relevant features and classifying samples. RF is particularly suitable for analyzing high-dimensional and complex data sets such as gene expression data, because it can handle a large number of variables and avoid overfitting. In addition, RF can provide a ranking of feature importance, which is valuable for identifying the genes most relevant to the results of interest. Therefore, in our study, we chose to use RF for feature selection and identify the most important genes associated with sepsis [24–28]. Although other algorithms such as xgboost, GLM, and NB are also popular in similar studies, we chose RF because of its advantages in processing complex datasets and providing feature importance rankings. Based on RF, this study screened the characteristic gene METTL16 as the most important gene in advanced sepsis. Like METTL3, the METTL16 gene also belongs to a class I methyltransferase that contains Rossmann fold of class I methyltransferases and uses S-adenosylmethionine (SAM) as the methyl donor. Moreover, METTL16 also contains other regions outside its methyltransferase domain, and these regions also interact with RNA substrates and may provide specificity. Intriguingly, METTL16 is commonly captured in the identification of mRNA binding proteins separated with polyadenosylated mRNA and endogenous interaction proteins, while the components of METTL3/14 complex are not captured. Although many METTL16 RNA interactants have been identified, only two of them have been confirmed as methylation substrates [29], which may indicate that METTL16 has other functions besides catalytic activity. Our study found that METTL16 gene was significantly positively correlated with the proportion of multiple immune cells, suggesting that METTL16 may promote the infiltration of immune cells in the occurrence and development of sepsis.

## Conclusions

In this study, the m^6^A-related genes differentially expressed in healthy subjects and advanced sepsis patients were obtained. Based on the RF model and correlation analysis, IGFBP1, IGFBP2, IGF2BP1, WTAP, and METTL16 were screened out to accelerate the occurrence and development of sepsis by regulating m^6^A methylation and promoting immune cell infiltration. These genes provide potential drug targets for the early detection, diagnosis, and treatment of sepsis.

## Supplementary Information

**Additional file 1: Table S1.** Identification of m6A clusters

**Additional file 2: Table S2.** m6A differential gene expression

## Data Availability

The datasets generated and/or analysed during the current study are available in the GEO [GSE175453] repository (https://www.ncbi.nlm.nih.gov/geo/query/acc.cgi?acc=GSE175453).

## Abbreviations

GEO: Gene expression comprehensive database
RF: Random forest
ssGSEA: Single-sample gene set enrichment analysis
ICUs: Intensive care units
m^6^A: N6 methyladenosine
PBMCs: Peripheral blood mononuclear cells
RBM15B: RNA binding motif protein 15B
IGFBP3: Insulin-like growth factor binding protein 3
IGF2BP1: Insulin-like growth factor 2 mRNA binding protein 1
ZC3H13: Zinc finger CCCH domain-containing protein 13
CBLL1: Casitas B-lineage lymphoma-transforming sequence-like protein 1
YTHDF1: YTH N^6^-methyladenosine RNA binding protein 1
HNRNPC: Heterogeneous nuclear ribonucleoprotein C
ELAVL1: Embryonic lethal-abnormal vision like protein 1
METTL3: Methyltransferase-like 3
RBMX: RNA-binding motif protein X-linked
PPR: Pentatricopeptide repeat
FTO: Fat mass and obesity-associated gene
WTAP: Wilms tumor 1-associated protein
YTHDC1: YTH domain containing 1
FMR1: Fragile X mental retardation type 1
HNRNPA2B1: Heterogeneous nuclear ribonucleoprotein A2B1
SVM: Support vector machine
ROC: Receiver operating characteristic
ANOVA: Analysis of variance
